# Are Standard Intra-Abdominal Pressure Values Different during Pregnancy?

**DOI:** 10.1371/journal.pone.0077324

**Published:** 2013-10-25

**Authors:** Florent Fuchs, Marie Bruyere, Marie-Victoire Senat, Emilien Purenne, Dan Benhamou, Hervé Fernandez

**Affiliations:** 1 Department of Obstetrics and Gynecology. Hôpital Bicêtre, Assistance Publique Hôpitaux de Paris, Le Kremlin-Bicêtre, France; 2 Inserm, Centre for research in Epidemiology and Population Health, Unit 1018, Reproduction and child development, Villejuif, France; 3 Paris-Sud University, Unit 1018, Villejuif, France; 4 Département of Anesthesia, Hôpital Bicêtre, Assistance Publique Hôpitaux de Paris, Le Kremlin-Bicêtre, France; University of Tennessee Health Science Center, United States of America

## Abstract

**Background:**

Measurement of intra-abdominal pressure (IAP) is an important parameter in the surveillance of intensive care unit patients. Standard values of IAP during pregnancy have not been well defined. The aim of this study was to assess IAP values in pregnant women before and after cesarean delivery.

**Methods:**

This prospective study, carried out from January to December 2011 in a French tertiary care centre, included women with an uneventful pregnancy undergoing elective cesarean delivery at term. IAP was measured through a Foley catheter inserted in the bladder under spinal anaesthesia before cesarean delivery, and every 30 minutes during the first two hours in the immediate postoperative period.

**Results:**

The study included 70 women. Mean IAP before cesarean delivery was 14.2 mmHg (95%CI: 6.3–23). This value was significantly higher than in the postoperative period: 11.5 mmHg (95%CI: 5–19.7) for the first measurement (p = 0.002). IAP did not significantly change during the following two postoperative hours (p = 0.2). Obese patients (n = 25) had a preoperative IAP value significantly higher than non-obese patients: 15.7 vs. 12.4; p = 0.02.

**Conclusion:**

In term pregnancies, IAP values are significantly higher before delivery than in the post-partum period, where IAP values remain elevated for at least two hours at the level of postoperative classical abdominal surgery. The knowledge of these physiological changes in IAP values may help prevent organ dysfunction/failure when abdominal compartment syndrome occurs after cesarean delivery.

## Introduction

Abdominal compartment syndrome (ACS) is the ultimate and most dreaded complication of elevated intra-abdominal pressure (IAP), and is associated with a reported mortality ranging from 50–100% [1], [2]. Intra abdominal hypertension (IAH) is defined by an IAP>12 mmHg, whereas ACS is defined by an IAP>20 mmHg, in the context of symptomatic organ dysfunction or failure [3] stemming from the cardiovascular, renal, mesenteric, central nervous, and musculoskeletal systems. The prevalence of these condition is highest in the post-surgical, critically injured, and trauma patient populations. Recent studies have demonstrated that ACS can also occur in a wide variety of medical and surgical conditions [4], [5]. Monitoring IAP has therefore become an important indicator of hemodynamic stability in intensive care units. Pregnancy is a particular condition where multiple factors such as obesity, preeclampsia or postpartum hemorrhage may lead to the over-diagnosis of ACS [6], [7], [8]. Few studies have therefore tried to establish normative values of IAP during pregnancy or in immediate postpartum according to various scenarios: supine, left lateral decubitus or leftward tilt; no anaesthesia or spinal anaesthesia, amongst others. Thus far, no clear references have emerged from these studies [7], [8], [9], [10], [11]. Establishing normative IAP values in pregnancy, and knowing the IAP threshold values that define IAH or ACS in pregnancy, may help obstetricians proceed to surgical decompression in case of persistently elevated IAP. The aim of this study was to prospectively assess normative values of IAP before and after elective cesarean section at term.

## Materials and Methods

A total of 70 patients were prospectively enrolled from January 2011 through December 2011 in a French tertiary care centre. All included patients gave their written consent to participate in the study after reading the consent form. The protocol, as well as the patient consent form was approved by an Institutional Review Board (Comité de protection des personnes Ile de France X) under the identification number 2010-A00984-35. Inclusion criteria were pregnant women at term (≥37 weeks’ gestation) with a singleton gestation that underwent an uneventful pregnancy and presented for scheduled elective cesarean delivery in the absence of labor. Exclusion criteria were patient’s age under 18, twin gestation, presence of labor, and any maternal medical complication that would potentially affect the IAP measurement such as gravid hypertension, preeclampsia, diabetes, nephritic syndrome, neurogenic or radiation bladder or abdominal masses. Maternal parameters were recorded. Gestational age was determined according to crown rump length measurement during first-trimester ultrasound. Patient consent was obtained before cesarean delivery. Participants were then transferred to the operating room, where spinal anaesthesia was administered and confirmed to be effective up to a T4 dermatome level. Drugs used in spinal anaesthesia were 10 mg of hyperbaric bupivacaine, 3 mcg sufentanil and 100 mcg morphine. Patients were then placed in the dorsal supine position, where a transurethral 16-Fr Foley catheter was inserted into the bladder, which was subsequently emptied under aseptic technique. According to the method described in the World Society of the Abdominal Compartment Syndrome guidelines [3], an intravenous 500 cc saline solution bag was then hung above the patient and placed into in a pressure bag inflated at 300 mmHg. Following, the solution bag was connected to transducer tubing that was flushed to remove air. The transducer system was also connected to the pressure monitoring cable and zeroed at the level of the pubic symphysis. The Foley catheter was clamped 2 centimetres distal to the port. The pressure tubing was then connected to the Foley catheter via an 18-gauge needle through the port on the catheter tubing. A 50-mL syringe was attached to the distal stopcock and filled with saline, and the stopcock turned off toward the patient. The operator then injected 25 mL of saline into the bladder and measurement of IAP was performed following 30–60 seconds, after pressure stabilization and end of respiratory expiration were ensured. IAP was measured before delivery in the operating room and then, every 30 minutes (M0, M30, M60, M90, and M120) during two hours in the postoperative recovery area. Time between spinal anesthesia and preoperative IAP measurement was approximately 10 minutes. Ten to fifteen minutes were required to transfer the patient from the operating room to the postoperative recovery area. Dermatome level of the residual effect of spinal anesthesia was evaluated, for the first post operative IAP (M0) measurement. During surgery, blood loss quantification was performed. Before discharging the patient from the postoperative recovery area, at M120, an ultrasound scan was performed to screen for the presence of blood in the pouch of Douglas and to quantify its level relative to uterine height.

Data are presented in mean (95%CI) or median [1^st^ quartile; 3^rd^ quartile] values according to normality of their distribution. In order to consider the non-independence of the data, we used linear mixed model to compare IAP measurements made at different times. Data was also analyzed using paired Student’s t-test for two groups mean comparison. Results were considered significant when p<0.05. Statistical analyses were performed with STATA software, v.11 (Stata Corporation, College Station, TX).

## Results

**Figure 1:**
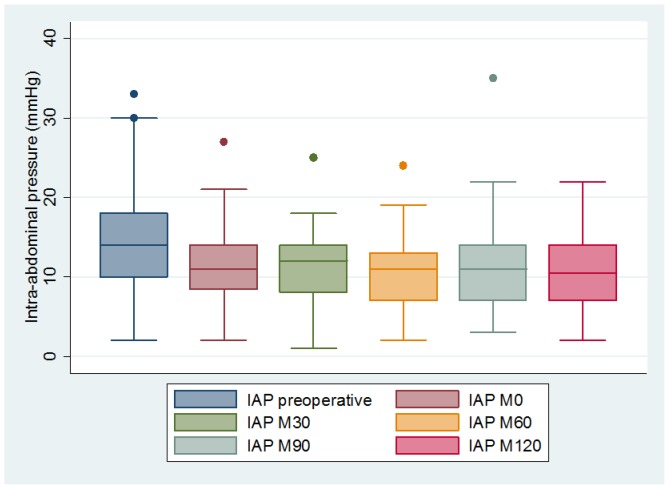
Intra-abdominal pressure before cesarean section (preoperative) and postoperative (every thirty minutes during two hours).

A total of 70 women were enrolled in the study. Patient characteristic, demographic data and IAP values are presented in Table 1. Mean maternal age was 34 years (95%CI: 27–41), median pre-pregnancy body mass index (BMI) was 26 [1^st^ quartile: 23.5; 3^rd^ quartile: 32.5], and average BMI immediately prior to surgery was 30.3 [27.1; 35.1]. Thirty-six percent of patients (n = 25) were classified as obese (pre-pregnancy BMI>30). The elective cesarean deliveries were performed at a median gestational age of 38.1 weeks. Reasons for cesarean deliveries included: repeat cesarean section (61%), breach or transverse presentation (31%) or placenta praevia (8%). Mean birth weight was 3210 g (95%CI: 2445–4215 g). None of the cesarean sections were complicated by post-partum hemorrhage or visceral injury. Cesarean section lasted on average 50 minutes (range 35–54 minutes). Median dermatome level at M0 was T10 [T9; T10]. IAP values followed Gaussian distribution (Shapiro-Wilk W test; p = 0.13). Preoperative IAP (14.2 mmHg (95%CI: 6.3–23)) was significantly higher than immediate (M0) (11.5 mmHg (95%CI: 5–19.7)) or delayed (M30, M60, M90, M120) postoperative IAP which was 11.2 mmHg (95%CI: 4.5–18), 10.8 mmHg (95%CI: 4.5–18), 11.2 mmHg (95%CI: 5–19) and 10.7 mmHg (95%CI: 4.5–18) respectively; p = 0.002. Indeed, IAP did not significantly change during the two postoperative hours (p = 0.2) (Figure 1). Obese patients (BMI>30) had significantly higher preoperative IAP levels than non-obese patients: 15.7 vs 12.4; p = 0.02. However, this difference disappeared after delivery, with several IAP measurements in the postoperative room remaining steady (p = 0.4). Patient’s age had no influence on the pre- or post-operative IAP. Seven women (10%) had a pre-operative IAP>20 mmHg, which is the usual threshold of ACS. For all of them, the IAP significantly decreased post-operatively and then remained stable for the two hour survey: 25.6 mmHg pre-operatively, 12.3 at M0 (p<0.001) and 12.5 at M120 (p = 0.8). Mean blood loss was 395 mL (95%CI: 70–950). Four women (6%) presented with blood in the pouch of Douglas (less than one third height of the uterus) without any influence on postoperative IAP value.

**Table 1:** Patient characteristic, demographic data and intra-abdominal pressure.

	n = 70
Age (year)	34 (27–41)
Pre pregnancy BMI* (kg/m^2^)	26 [23.5; 32.5]
BMI at cesarean section (kg/m^2^)	30.3 [27.1; 35.1]
Gestational age at cesarean (weeks)	38.1 [38; 39]
Infant birth weight (gramms)	3210 (2445–4215)
Intra-abdominal pressure (mmHg)	
Preoperative	14.2 (6.3–23)
Postoperative M0	11.5 (5–19,7)
Postoperative M30	11.2 (4.5–18)
Postoperative M60	10.8 (4.5–18)
Postoperative M90	11.2 (5–19)
Postoperative M120	10.7 (4.5–18)

* Body mass index

Data are presented as mean (95%CI) or median [1^st^ quartile; 3^rd^ quartile]

## Discussion

In this study, we found that intra-abdominal pressure measured prospectively in a group of uncomplicated pregnancy undergoing elective scheduled cesarean delivery at term was significantly higher in the pre-operative period and then recovered rapidly in the post-operative period to a level that sustained for two hours.

In the USA as well as in the UK, few studies have shown that pregnancy was a particular condition where the use of critical care services was required in up to 1–3% [12], [13]. The most frequent reasons for admission were maternal hypertension and postpartum hemorrhage. We also know from an intensive care unit (ICU) study that IAH is present in 38% of patients upon admission, and is an independent predictor of mortality [4] In the same study the overall prevalence of IAH reached 59%, and 8% for ACS. Therefore, monitoring IAP is becoming a standard of care in ICU, especially for patients admitted with respiratory failure, increased abdominal contents or massive fluid resuscitation [3]. However, normal levels of IAP in pregnant women have been poorly studied so far.

There is a debate as to how IAP should be measured in pregnant women at term, particularly regarding maternal position, type of maternal anaesthesia, and breathing condition. We chose to measure IAP in fully supine, under spinal anaesthesia and at end-expiration for several reasons. The main reason is that this maternal positioning follows the current recommended guidelines of IAP measurement as described by the World Society of the Abdominal Compartment Syndrome (WSACS) [3]. Moreover, it is known that lateral tilt does not provide fetal/neonatal benefits in APGAR scores or umbilical artery pH, and is therefore not mandatory [14], [15]. Chun et al. [6] compared supine and left lateral tilt positioning for measuring IAP. Supine positioning appeared to be associated with higher IAP value (10.9 mmHg versus 8.9 mmHg) probably related to the weight of the gravid uterus on the bladder. In another study, Sugerman [11] compared supine and left lateral decubitus positioning. Similarly, supine position was associated with an increase in the IAP value (25 mmHg versus.23 mmHg). However values of IAP in supine position were very different in both studies,this is probably related to a smaller number of patients and unclear methods in the Sugerman study (only 5 patients included), giving imprecise evaluations. Al-Khan et al. [8] conducted a large study with 100 patients recruited in a very short period, 2 months, and found extremely high IAP values (22 mmHg) for scheduled cesarean section. However, their methodology could have created some bias leading to an overestimation of IAP values. Indeed, as previously noticed by Chun et al.[6], in Al-Khan study [8], degree of lateral tilt was not specified, dermatome level of spinal anaesthetic was unknown leading to possible under relaxed abdominal wall muscle, and 50 mL instead of 25 mL was instilled in the bladder for the measure. Even with a high number of patients, that could give powerful conclusion, this derived methodology renders the results inappropriate. These findings confirm the current need to standardize the methodology used for measuring IAP values in pregnancy before interpreting its results.

Our study enabled to clarify three particular question regarding IAP in pregnant women. First, physiological IAP in pregnant women is increased compared to normal IAP values in non-pregnant women, which is about 5–7 mmHg [3]. This increase takes place without any visceral impairment, as it occurs gradually during the 9 months’ of pregnancy. Second, we confirmed the influence of obesity on IAP values. It has previously been reported that obesity increases the baseline IAP up to 9–14 mmHg [16] in morbidly obese non pregnant women. The same phenomenon was observed in our study were IAP was significantly higher in obese pregnant women than in non obese. This correlation between BMI and IAP has also been reported previously [7], [8]. Third, we found that IAP remained steady after delivery, but at an elevated level (around 11 mmHg) relative to non-pregnant women (5–7 mmHg). However, this value is in accordance to standard value of IAP following uncomplicated abdominal surgery (10–15 mmHg) [17], [18]. Many factors have been described to explain this physiological increase after surgery [18], but in obstetrics, the two main etiologies are probably the persistent increase in uterine size, and the cesarean section itself. Reasons for pathological postoperative IAP increases are digestive impairment, visceral edema or small intestine dilatation, rather than intra-abdominal effusion (except in the case of massive hemoperitoneum) [3]. This may in part explain why ACS frequently occurs in intensive care units after long lasting abdominal surgery with hemodynamic shock or bowel manipulation, rather than short surgery without visceral wounds, such as in cesarean sections. This post operative level (11 mmHg) may therefore correspond to the physiological post partum value and could be important to define pathological increase of IAP. As it is regularly performed for ICU patients, we think that every pregnant woman admitted to ICU after delivery should be monitored for IAP.

A limitation of our study is that dermatome level had not been evaluated during the two hours post operative survey. Indeed, only M0 dermatome level was recorded. This could have created a bias because postoperative IAP value could be related to the level of anesthesia rather than to the effect of delivery in pregnant women. However, as the usual reported duration of action of bupivacaine is 90–120 minutes, we believe that it could have influenced IAP value up to the M60 measurement, considering the timing of cesarean section and of patient transfer to post operative recovery area. As we did not observe any significant change of IAP before and after M60, we therefore believe that the influence of spinal anesthesia may be relatively low. To confirm our data, a specific evaluation of IAP before and after spinal anaesthesia administration is warranted as well as an evaluation of dermatome level during the two hours in the postoperative area.

In conclusion, the range of IAP in women measured before elective cesarean is significantly higher than after cesarean delivery. The value of IAP after delivery remained steady during at least two hours at the level of postoperative classical short abdominal surgery. The knowledge of these physiological changes in IAP values may help prevent organ dysfunction/failure when abdominal compartment syndrome occurs. Further studies are needed to confirm our results, both for clinical situations requiring IAP measurements after delivery and also regarding the post operative threshold for IAP where an additional treatment is needed.
